# Toward the future of next-generation dairy foods—A processing perspective and economic analysis*

**DOI:** 10.3168/jdsc.2024-0727

**Published:** 2025-05-12

**Authors:** Gulustan Ozturk, Charles F. Nicholson, Richard W. Hartel

**Affiliations:** 1 Department of Food Science, University of Wisconsin–Madison, Madison, WI 53706; 2 Department of Agricultural and Applied Economics, University of Wisconsin–Madison, Madison, WI 53706

## Abstract

There is a need for sustainable food production and processing that reduces resource use and increases the availability of nutritious, innovative, and sustainable food. A coordinated, multisectoral approach across the food supply chain is essential to address global food and nutrition insecurity. The dairy industry produces abundant bioactive compound streams that can be examined for their valuable functionalities. Whey protein phospholipid concentrate (WPPC) is a byproduct of whey protein isolate production and a source of many bioactive compounds, including the milk fat globule membrane (MFGM). Milk fat globule membrane has unique functionalities, such as promoting brain development, modulating the immune system, improving the growth of desirable gut bacteria, and reducing inflammatory and metabolic diseases. We recently demonstrated that WPPC contained relatively higher levels of MFGM proteins, representing 23% of the total protein and phospholipids accounting for 20% of the total fatty acid pool. In this symposium review, we use WPPC as a case study to demonstrate dairy streams with the potential to be used as cutting-edge ingredients to develop next-generation dairy foods that are nourishing, innovative, and sustainable. Additionally, we assessed the economic costs and benefits of higher-valued uses of WPPC (e.g., in human food applications rather than as animal feed), which complements the discussion of the functionality of WPPC in various applications.

## Highlights

• Whey protein phospholipid concentrate (WPCC) is a promising ingredient for next-generation dairy foods with health benefits.
• Economic analysis suggests WPPC's higher value potential in human food applications.
• Unique composition of WPPC enhances functionality in various food products.
• Sustainable dairy processing can help combat global food and nutrition challenges.

## Graphical Abstract

**Figure:**
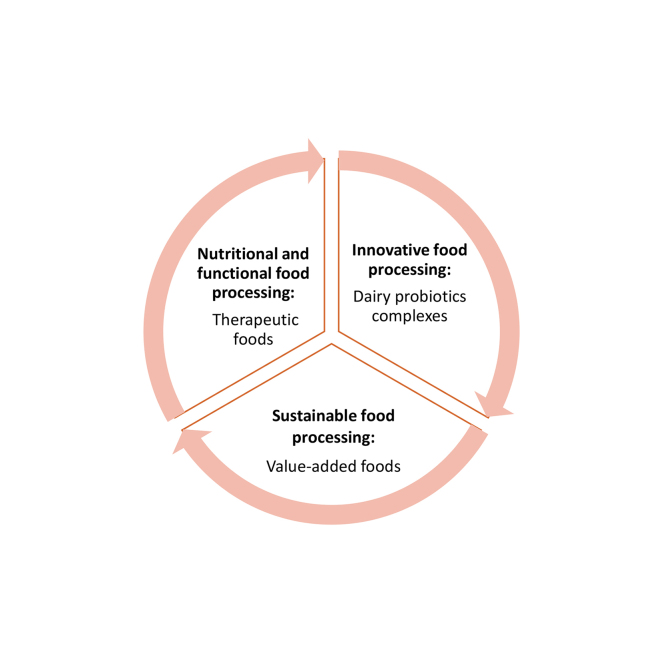
**Summary:** As the dairy industry evolves—shaped by advances in processing, consumer interest in health and sustainability, and product innovation—bioactive compounds hold the potential to be incorporated into the next generation of dairy foods, directly, or through dairy-inspired formulations.

The world is currently navigating significant challenges and opportunities in the realm of food and nutrition. Obesity, affecting ~2 billion people worldwide, imposes a significant strain on healthcare systems. At the same time, more than 2 billion individuals experience hidden hunger, where despite sufficient calorie intake, they are deficient in vital micronutrients (WHO, 2024). Addressing these issues requires a focus on environmental sustainability, with both food production and processing needing to reduce resource use while producing greater quantities of nutritious, innovative, and sustainable food. A coordinated, multisectoral approach across the entire food supply chain is necessary to tackle global food and nutrition insecurity.

Within the broad spectrum of food production, dairy holds a promising position to contribute to addressing global challenges and trends. Dairy products have the potential to emerge as one of the most beneficial categories in the human diet. Lactation offers a valuable model for developing future research strategies aimed at creating a food supply that is both nourishing and safe (German et al., 2022). Human milk serves as a basis for these developments. Milk is a complex fluid that provides complete postnatal nutrition for mammalian infants. It contains essential nutrients and abundant bioactive compounds such as antibodies, antimicrobial peptides, oligosaccharides, and the milk fat globule membrane (**MFGM**). These bioactive compounds are capable of modulating gut microbial composition, supporting gut health, and reducing inflammation associated with dysbiosis (Pennisi, 2019). The MFGM is a complex structure that originates from the mammary gland epithelium and comprises ~40% lipids (e.g., phospholipids) and 60% proteins (e.g., glycoproteins). These glycosylated proteins perform specialized functions for health (Dewettinck et al., 2008). Several glycosylated bioactive proteins are embedded in the MFGM, including butyrophilin, xanthine oxidase, glycosylation-dependent cell adhesion molecule 1 (GLYCAM-1), mucin 1 and 15, lactadherin, adipophilin, fatty acid-binding protein heart, polymeric immunoglobulin receptor, and platelet glycoprotein 4. The lipid fraction of the MFGM is mainly composed of polar phospholipids, such as phosphatidylcholine (**PC**), phosphatidylethanolamine (**PE**) sphingolipids (**SL**), and sphingomyelin (**SM**). Each of these compounds has unique functionalities, such as promoting brain development, modulating the immune system, exhibiting antimicrobial properties, improving the growth of desirable gut bacteria, and reducing inflammatory and metabolic diseases (Favre et al., 2011; Lordan and Zabetakis, 2017; Brink and Lönnerdal, 2018).

In this symposium review, we will focus on whey protein phospholipid concentrate (**WPPC**) as a dairy stream with potential as a cutting-edge ingredient for developing innovative, nourishing, and sustainable next-generation dairy foods (see Graphical Abstract). We will begin by discussing the composition, functionality, and processing and fractionation of WPPC and next-generation dairy foods. We will then evaluate the economic costs and benefits of utilizing WPPC in higher-value applications, such as human food products, rather than merely using it as animal feed. This analysis will complement our discussion on the functionality of WPPC in various applications.

The dairy industry produces abundant bioactive compound streams that can be examined for their valuable bioactive compounds. The development of novel products from whey has led to the creation of new coproducts. During the manufacturing of whey protein isolate (**WPI**), whey protein concentrate is microfiltered, separating most of the whey proteins from the residual fat. Consequently, there has been a substantial accumulation of a coproduct known as WPPC. The American Dairy Products Institute (**ADPI**) has established a marketing standard sheet for WPPC, stating that WPPC composition should consist of a minimum of 50% protein (dry basis) and 12% fat, a maximum of 8% ash and 6% moisture, and a pH between 5.7 and 7.5 (ADPI Product Standards, 2023). Recent studies analyzed the average total solids, protein, and fat content in the liquid and powder WPPC provided by several dairy manufacturers, including 2 lots produced 6 mo apart by each supplier. Fat and protein values comply with the WPPC ingredient standard published by the ADPI; however, significant differences were observed between lots in all WPPC composition categories, and the greatest variations were observed in the fat and protein content. These findings demonstrated that WPPC composition was highly variable between suppliers and lots (Ozturk et al., 2022).

In 2023, the United States produced 140 million pounds (63.5 million kilograms) of WPI and 77 million pounds (34.9 million kilograms) of WPPC, with WPPC primarily used in low-value products, including animal feed (ADPI, 2023). In parallel, the accumulation of WPPC calls for the identification of suitable avenues for its valorization (Ozturk et al., 2022).

Although the gross composition of WPPC is known, detailed characterization was not available until recently. It has been demonstrated that WPPC is uniquely enriched in a subset of bioactive proteins derived from MFGM (Ozturk et al., 2022). Whey protein phospholipid concentrate contains higher levels of bioactive O-linked and N-linked MFGM-bound glycoproteins, accounting for 23% of total proteins compared with 1% to 2% in bovine milk and 1% in human milk. In detail, the most abundant MFGM protein was GLYCAM-1, at 7% abundance, followed by butyrophilin (5%), lactadherin (4.45%), xanthine oxidase (2.25%), and fatty acid-binding protein heart with an abundance of less than 2%. β-lactoglobulin (30%) is still the predominant milk protein. Serum albumin was the second most abundant milk protein (11%) in WPPC, followed by lactoferrin (3.80%). Whey protein phospholipid concentrate is a rich source of polar lipids, constituting 20% of the total lipid pool. Among those, PC and PE were the most abundant phospholipids (a concentration of ~9 mg/g each as of their fatty acid content). PS and PI were less abundant, at 2.1 to 3.3 mg/g, followed by SL containing SM at 1.0 mg/g as of their fatty acid content. Glycosylated proteins and glycolipids in MFGM have sialic acids, which are crucial for brain development and gut microbial populations in infants. Major sialic acid compounds, such as N-acetylneuraminic acid at 1.51% ± 0.02% by weight, with trace amounts of N-glycolylneuraminic, are also present. Whey protein phospholipid concentrate also contains anti-inflammatory oxylipins derived from eicosapentaenoic acid and docosahexaenoic acid, suggesting it is a natural source of anti-inflammatory compounds (Ozturk et al., 2022).

Whey protein phospholipid concentrate is a valuable ingredient due to its high content of protein, fat, and phospholipids, making it suitable for a variety of functional applications. These include emulsification, foamability, and water-holding capacity, which can be used in numerous value-added food products, such as protein bars, processed cheese, ice cream, beverages, frozen yogurt, and salad dressings. A study explored different blends of delactosed permeate with WPPC as a protein and fat source in ice cream (Bund and Hartel, 2013). The study compared these blends with a control ice cream that used nonfat dry milk as the protein source. Results indicated that although all ice cream samples had similar mean ice crystal sizes, the WPPC and delactosed permeate blends exhibited the highest melt rate and reduced fat destabilization compared with the control. Another investigation assessed the effectiveness of WPPC in ice cream, caramels, and cake (Levin et al., 2016). The study found that WPPC could replace synthetic chemical emulsifiers in ice cream, substitute sweetened condensed milk in caramels, and act as a partial replacement for whole eggs in cakes. The ice cream produced with WPPC had a lower overrun and higher melt rate but similar ice crystal and air cell sizes compared with the control. In caramels, using WPPC resulted in increased cold flow and reduced hardness and stickiness, and in cakes, WPPC produced yields comparable to those of control cakes made with whole eggs, though with lower volume and contour. The variability in the composition of WPPC powders was noted to affect its functionality in different food products. Consequently, isolating the phospholipids from WPPC could potentially produce 2 distinct ingredients—protein and phospholipids —that offer greater functionality than WPPC in its original form.

Beyond its functional properties, WPPC holds significant potential for nutritional applications, particularly in infant formula. Human milk serves as a model for developing infant formula, and phospholipids are crucial nutrients that contribute to growth and brain development in infants (Hinde and German, 2012). The phospholipids found in bovine milk, similar to those in human milk, vary in concentration but share key characteristics, making them valuable ingredients in infant formula. Given its high MFGM content, WPPC presents a promising resource for extracting and applying these components in similar products. Recently, ADPI introduced standards for infant formula–grade WPPC, which include specific requirements for this application. According to ADPI (2023), WPPC for infant formula should contain at least 50% protein (dry basis), 12% fat, and 4% total phospholipids. The bioactive compounds present in the WPPC offer promising opportunities as innovative ingredients. Developing methods to achieve high purity and sufficient quantities of these bioactive compounds is crucial for enabling in-depth mechanistic studies.

Heat treatments are critical to milk processing, primarily to ensure microbial safety (Bylund, 2003). Bovine milk can be HTST pasteurized, where milk is heated at 72 °C for at least 15 s, or at higher temperatures for shorter times (UHT), where milk is heated at 135 to 150 °C for 2 to 6 s. Some small manufacturers use the batch pasteurization process, where milk is heated to 63 °C for 30 min (Chandan, 2011). However, these treatments also significantly alter the physical, chemical, and biological properties of milk components (Ozturk et al., 2019, 2024; Kim et al., 2023). For instance, heat can modify the structure of whey proteins, particularly those containing free sulfhydryl groups. The denaturation and aggregation of whey proteins have long been areas of interest in the dairy industry because they significantly affect processing performance, as well as the functionality and quality of the final product. The denaturation and aggregation of whey proteins are typified by redistributing the intramolecular forces between proteins, leading to unfolding and loss of native protein structure (Anema, 2008). Whey protein aggregates such as β-LG aggregates accelerated membrane fouling during microfiltration and ultrafiltration due to covalent thiol-disulfide reactions, whereby aggregates acted as nucleation sites for the buildup of the fouling layer, concluding that even low levels of aggregation (5% aggregated β-LG) contributed to a considerable reduction in membrane flux (Steinhauer et al., 2015). Research has shown that the production of WPPC involves the formation of complexes between whey proteins and the MFGM due to heat. Swaminathan et al., 2021 explored various chemical pretreatment methods, such as isoelectric precipitation, thermocalcic aggregation, and calcium-induced aggregation of whey proteins, to separate phospholipids from WPPC (Swaminathan et al., 2021). Although these approaches had previously succeeded in removing fats from cheese whey and buttermilk (Rombaut and Dewettinck, 2006; Rombaut et al., 2007), they were ineffective with WPPC solutions, likely due to the close association between protein and fat in WPPC, which forms large protein aggregates that trap fat. Confocal laser scanning microscopy confirmed the presence of extensive protein-fat aggregates, creating a complex matrix that makes phospholipid extraction challenging with basic chemical methods. A different approach involves enzymatic hydrolysis of the residual proteins in WPPC. Studies using enzymes like alcalase demonstrated a decrease in major protein bands over time, along with smaller peptide bands appearing as hydrolysis continued. Despite 1 h of hydrolysis, confocal microscopy and particle size analysis indicated that protein aggregates persisted, suggesting that additional enzymes may be necessary to fully break down the protein aggregates and further concentrate phospholipids in WPPC (Swaminathan et al., 2023). Thus, it is important to investigate the changes that occur within the WPPC matrix during thermal processing. Understanding how thermal treatments alter the WPPC matrix is therefore essential. Specifically, gaining insights into how whey proteins are affected by different heat treatments can improve the efficiency of isolating valuable compounds such as whey proteins, MFGM proteins, and phospholipids, which have significant nutritional and economic benefits.

The dairy industry is undergoing significant transformation, driven by advancements in processing technologies, consumer demands for health and sustainability, and innovations in product development. The current health crisis, marked by increasing rates of diabetes, metabolic syndrome, and inflammatory conditions, highlights the need for precision nutrition (Bailey and Stover, 2023). Diet remains a powerful, modifiable factor in promoting human health, underscoring the urgency for evidence-based public health strategies to combat high rates of nutrition-related chronic illnesses (Bailey and Stover, 2023). Milk contains bioactive compounds and nutrients capable of modulating gut microbial composition, supporting gut health, and reducing inflammation associated with dysbiosis (Pennisi, 2019). This review focuses specifically on MFGM bioactive compounds. Our research identified WPPC as a rich source of MFGM, making it a valuable component for human health applications (Ozturk et al., 2022). The next generation of dairy foods could incorporate these dairy components, either explicitly or through a dairy-inspired approach.

Despite its fascinating composition, the economic and nutritional value of WPPC has yet to be thoroughly explored. Whey protein phospholipid concentratehas been recognized as an excellent source of membrane-related phospholipids, which have the potential for various nutrition-based applications. Assessment of the economic costs and benefits of higher-valued uses of WPPC (e.g., in human food applications rather than as animal feed) complements the discussion of the functionality of WPPC in various applications. A key condition for expanded and higher-value use of WPPC is the value proposition. A value proposition is a statement that identifies clear, measurable and demonstrable benefits a customer receives when buying a product or service (Otakanon et al., 2020). This information should convince customers that the product or service is better than others currently available for a specific application. There must be a sustained value proposition for higher-value uses of WPPC to support growth, where “sustained” means likely to persist for a sufficiently long period of time to result in a positive return on investment. The value proposition for WPPC depends on the costs and benefits of current or potential manufacturers of WPPC and current or potential end users of WPPC. For manufacturers, documentation is required for the investments in plant and equipment to manufacture WPPC and the operating and logistics costs associated with assembling raw materials, making product and distributing it to customers. For end users, the benefits may comprise improved functionality of WPPC compared with other ingredients, reduced costs, or both. This necessitates information on costs to end users of WPPC, and any potential cost savings. Many factors will affect the future potential for WPPC, including differences by region (location) in raw material availability and relevant costs such as labor, utilities and transportation. Here, we highlight the process to evaluate WPPC in alternative uses, illustrate the information required, and, provide ranges of possible values for the WPPC value proposition.

The manufacture of WPPC is associated with producing WPI. A typical plant would process 1 to 1.5 million kilograms of separated whey per day. Industry sources indicate that the cost range for required equipment (ultrafiltration and microfiltration system, dryer, packaging line) would be $34 to $40 million, not including any additional construction costs. The calculated annual yields of WPPC from average daily volumes of 1 to 1.5 million kilograms of separated whey processed per day are estimated to range from 0.8 to 1.7 million kilograms (Table 1).

**Table 1:** Calculation of per-unit manufacturing costs of WPPC produced in a facility processing 1.0 or 1.5 million (mil) kg of separated whey per day

Calculation element	Unit	Quantity of separated whey processed per day	
		1 mil kg	1.5 mil kg
Processing days per week	d/wk	6	6
Quantity processed per week	mil kg/wk	6.00	9.00
Quantity processed per year	mil kg/yr	312.00	468.00
Proportion solids in separated whey^1^	proportion	0.06	0.06
Quantity of solids processed per year	mil kg/yr	18.72	28.08
Proportion of protein in whey solids^1^	proportion	0.1025	0.1025
Protein quantity processed per year	mil kg/yr	1.92	2.88
Proportion of protein in separated whey allocated to WPPC^1^	proportion	30%	30%
Quantity protein in WPPC	mil kg/yr	0.58	0.86
Protein content of WPPC			
Low value of range for protein content	proportion	0.50	0.50
High value of range for protein content	proportion	0.70	0.70
Quantity of WPPC produced			
Low value of range for protein content	mil kg/yr	1.15	1.73
High value of range for protein content	mil kg/yr	0.82	1.23
Annual operating cost^1^	$/yr	500,000	500,000
Equipment investment costs			
Initial investment costs^1^	$	33,500,000	40,000,000
Useful life of equipment	yr	25	25
Weighted average cost of capital^2^	%/yr	0.06	0.06
Annual economic cost of plant and equipment^3^	$/yr	2,649,387	3,163,447
Production cost per unit WPPC^4^			
Low value of range for protein content	$/kg	0.82	0.64
High value of range for protein content	$/kg	1.15	0.89

1 Information from industry key informants.

2 Source: Damodaran (2024); reported for the food processing category.

3 Annualized economic cost of plant and equipment including opportunity costs calculated using the PMT function in Microsoft Excel to calculate annualized cost based on initial investment cost and interest.

4 Cost per unit assuming allocation of total costs based on proportion of protein in the separated whey retained in WPPC, 30%.

We calculate the annual economic cost of plant and equipment, assuming a useful life of 25 years for the plant and equipment, and allocate the annualized equipment costs and operating costs to the products based on their proportion of protein in the separated whey (30% to WPPC, 70% to WPI). Equipment costs include an interest component at the weighted average cost of capital for food processing, which accounts for either actual cash interest payments (if the investment is funded by borrowing) or the economic opportunity cost of capital (if funded by equity). Operating costs for WPPC include inputs such as energy, labor and supplies and administrative costs (e.g., storage and overhead). Annual costs are approximately $500,000 per year for either 1.0 or 1.5 million kilograms of separated whey processed per day. Depending on the quantities processed and the assumptions about the proportion of protein in WPPC, costs of WPPC manufacture are estimated to range from $0.64/kg to $1.15/kg.

Separated whey needs to be assembled at a processing facility to provide the raw material for WPI and WPPC manufacturing. A WPPC facility may be co-located with a cheese plant, but this is not always the case, and it is not uncommon to source separated whey from multiple cheese plants. This implies logistics costs for transportation, loading and unloading, and for storage. Transportation costs for separated whey often can be estimated as a function of distance. Stephenson and Nicholson (2022) indicated that sourcing separated whey from a cheese plant 25 km away would cost about $0.009/kg of separated whey, or $0.003/kg WPPC with an allocation of 30% to WPPC. With a WPPC yield of 0.004 kg per kilogram separated, this implies inbound transportation costs of $0.79/kg. The cost of separated whey used as input can vary widely with market conditions and individual contractual arrangements. Nicholson and Stephenson (2015) estimated separated whey market values as $0.01/kg. Allocating 30% of this cost to WPPC results in a cost comparable to inbound transportation, about $0.90/kg WPPC. Stephenson and Nicholson (2022) estimated the cost of transporting dried WPPC 100 km would equal about $0.03. The landed cost to a WPPC buyer would include the product cost paid to the WPPC manufacturer plus the transportation costs to deliver the product to its intended use location. When all the costs of delivering the product to the buyer are included, this is referred to as the “landed cost” of the product. We estimate a range of values for landed cost for WPPC from $2.36 to $2.87/kg.

The value proposition for potential end users of WPPC requires the identification of potential uses of WPPC in human food (with similar or improved functionality) and the assessment of cost savings. Limited information has been published to date about both functionality and cost savings. Levin et al. (2016) noted that WPPC and DLP can be utilized as functional dairy ingredients at a lower cost in ice cream and cake but not in chewy caramel. We evaluate one example first discussed by Levin et al. (2016) for the use of WPPC in the cake. They evaluated 6 alternatives that included WPPC in cake ingredients to compare with conventional formulations, with WPPC as a substitute for eggs. Using recent cost information for each of the ingredients from selected sources, we evaluated the ingredient cost per 100 kg and the cost per kg of cake product. Assuming a value for WPPC equal to landed delivery costs of $2.36/kg, the use of WPPC in cake production had ingredient costs per 100 kg as much as $11 lower than the control, or as much as $0.09 per kg of cake (Table 2). These correspond to ingredient cost savings of 6% to 9% per kg of cake, which would seem sufficiently large to attract the interest of commercial bakers.

**Table 2:** Ingredient costs of control and 6 alternatives with WPPC in cake baking^1^

Cake ingredient	Ingredient cost, $/kg	Control	WPPC from A	30DLP: 70WPPC A	WPPC from B	30DLP: 70WPPC B	WPPC from C	30DLP: 70WPPC C
Water	0.02	0.55	0.76	0.75	0.76	0.75	0.76	0.75
Sugar	1.00	27.45	27.73	27.48	27.64	27.34	27.70	27.43
Flour	0.64	14.95	15.10	14.96	15.05	14.89	15.08	14.94
Shortening	3.01	30.37	30.67	30.40	30.58	30.25	30.64	30.37
Double-acting baking powder	3.50	4.90	4.93	4.90	4.93	4.86	4.93	4.90
Salt	0.72	0.43	0.44	0.43	0.43	0.43	0.44	0.43
Vanilla	20.77	10.38	10.59	10.38	10.59	10.38	10.59	10.38
Eggs	1.33	16.27	0.00	0.00	0.00	0.00	0.00	0.00
WPPC from A	2.36	0.00	4.96	4.96	0.00	0.00	0.00	0.00
WPPC from B	2.36	0.00	0.00	0.00	5.71	5.71	0.00	0.00
WPPC from C	2.36	0.00	0.00	0.00	0.00	0.00	5.22	5.22
DLP	0.62	0.00	0.00	0.56	0.00	0.66	0.00	0.58
Total cost, 100 kg	—	105.30	95.19	94.27	95.70	94.62	95.37	94.43
Weight, kg	—	89.70	88.50	88.80	88.40	86.40	88.20	87.30
Cost per kg	1.17	1.08	1.07	1.08	1.10	1.08	1.09	1.17
Change in costs, %		—	−9.6	−9.9	−9.1	−9.5	−9.4	−9.8
Change in costs per kg, %		—	−8.4	−9.0	−7.8	−6.1	−7.9	−7.3

1 Source: Author calculations based on ingredient combinations evaluated by Levin et al. (2016) and the value calculations above. The WPPC cost is assumed to be the calculated landed cost value of $2.36/kg. A: manufacturer A; B: manufacturer B; C: manufacturer C; DLP = delactosed permeate; WPPC = whey protein phospholipid concentrate; 30DLP:70WPPC: blend consists of 30% DLP and 70% WPPC.
